# Two Dutch Examples of Acute Geriatric Community Hospitals for Older Adults: A Comparative Process Evaluation

**DOI:** 10.5334/ijic.10214

**Published:** 2026-09-08

**Authors:** Eline Kroeze, Laurien Kanis, Gercora Hoitinga, Susanne Smorenburg, Janet MacNeil Vroomen, Anneke van Vught, Bianca Buurman

**Affiliations:** 1 Department of Internal Medicine, Section of Geriatric Medicine, Amsterdam UMC, University of Amsterdam, Amsterdam, The Netherlands; 2 Department of Medicine for Older People, Amsterdam UMC, Vrije Universiteit Amsterdam, Amsterdam, The Netherlands; 3 Amsterdam Public Health, Aging Later Life, Amsterdam, The Netherlands; 4 Department of Surgical Specialties, Canisius Wilhelmina Hospital, Nijmegen, The Netherlands; 5 Department of Emergency Medicine, Amsterdam UMC, The Netherlands; 6 Department of Internal Medicine, Section of Geriatric Medicine, Amsterdam UMC, The Netherlands; 7 University of Amsterdam, Amsterdam, The Netherlands; 8 Dutch Healthcare Authority (NZa), Utrecht, The Netherlands; 9 Canisius Wilhelmina Hospital, Nijmegen, The Netherlands

**Keywords:** acute geriatric unit, community hospital, intermediate care, process evaluation, RE-AIM, older people

## Abstract

**Background:**

Older adults with frailty are at high risk of adverse outcomes during hospitalisation. To address this, two Acute Geriatric Community Hospitals (AGCH1 and AGCH2) were established in the Netherlands. This study examined similarities and differences in setting, staffing, care pathways, patient populations, outcomes, and improvement opportunities in the first quarter of 2024.

**Methods:**

We conducted a mixed-methods evaluation using questionnaires (n = 2), documents (n = 10), case vignettes (n = 20), case vignette surveys (n = 7), and focus groups (n = 2). Quantitative data on patients admitted between January 1 and March 31, 2024, (n = 161) were collected in aggregated form via the questionnaires and analysed descriptively; qualitative data were analysed thematically.

**Results:**

Both sites implemented the core model elements but varied in setting and staffing. Patients showed high multimorbidity with respiratory conditions and infections as common diagnoses. Across ten case vignettes per AGCH, experts reported variation in referral appropriateness. Mean length of stay was shorter at AGCH2 (9.0 vs. 12.5 days), while less patients were discharged home (51.7% vs. 70.3%). Focus groups highlighted seven improvement areas: referral, expectation management, discharge planning, IT integration, funding, and evening/night/weekend care.

**Conclusions:**

The two AGCHs appeared to have largely similar care pathways, patient populations, and improvement opportunities, but differed in context, staffing, and outcomes. Sustainable implementation requires continuous learning, clear referral pathways, and skill mix review; national scale-up demands effectiveness studies, role delineation, and defining core versus adaptable model elements.

## Introduction

Countries worldwide struggle to meet the growing and complex care needs of ageing populations [1]. In the Netherlands, healthcare reforms in 2006 and 2015 aimed to support ageing-in-place, resulting in more older adults with frailty living at home [2]. Approximately 17% of adults aged 65+ living at home are considered frail, involving decline in physical, cognitive, social, and psychological domains [3,4]. Hospitalisation for this group carries high risks for adverse outcomes, including early unplanned hospital readmissions, functional decline and potential overtreatment in the final months of life [5,6,7,8].

To mitigate these risks and support independent living in the community, Acute Geriatric Units (AGUs) have emerged as a promising alternative to regular hospital care. AGUs provide hospital treatment for older patients in a community hospital, intermediate care, or nursing home setting when there is no major diagnostic uncertainty and the acute event or exacerbation is mild or moderate [9]. A systematic review and meta-analysis showed AGUs improve clinical and process outcomes: AGU care is associated with a 21% reduction in functional decline at six-month follow-up and a 6% increase in the likelihood of living at home at three-month follow-up [10].

**Figure 1:**
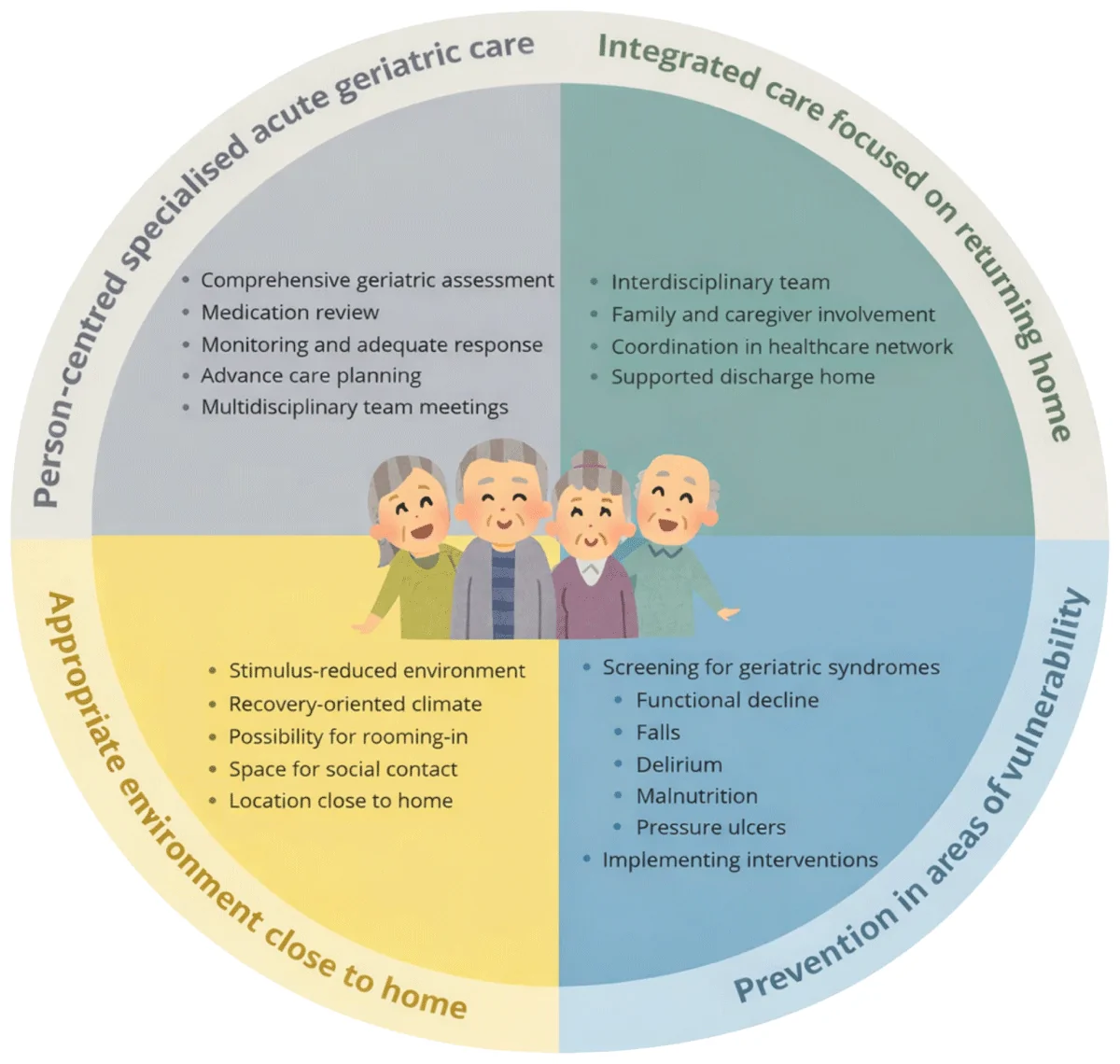
Core model elements of the Acute Geriatric Community Hospital (AGCH).

Building on these principles, the Acute Geriatric Community Hospital (AGCH) was introduced in the Netherlands in 2018 as a context-specific adaptation of the AGU model. The AGCH is an integrated care model that bridges hospital and intermediate care services across organisational boundaries. It provides acute geriatric care in a home-like setting with four core model elements: 1) person-centred specialised acute geriatric care; 2) integrated care focused on returning home, 3) prevention in areas of vulnerability; and 4) appropriate environment close to home (see Figure 1). Evaluations of the first AGCH showed promising outcomes. A prospective cohort study reported a 61% lower risk of 90-day readmission or death among AGCH patients compared to a historic hospital cohort [11]. Additionally, patient satisfaction was high [12], and delirium developed in 8% (18/214) of patients during AGCH admission, compared with 15% in historical hospital control groups [13].

A second AGCH opened in 2020 and differs in setting from the first AGCH. While the first AGCH operates in a skilled nursing facility and is coordinated by a hospital geriatrician, the second AGCH operates in an intermediate care ward within a general hospital and is coordinated by an elderly care physician (ECP; see Supplementary File 1). These contextual differences have raised questions among providers, insurers, and policymakers about whether the model is implemented as intended and whether outcomes are comparable. To date, it remains unclear how contextual and organisational differences between the two AGCHs influence implementation, care processes, and outcomes. This limits understanding of the AGCH model’s adaptability, effectiveness and scalability. Therefore, this study aimed to examine similarities and differences between the two AGCHs in setting, staffing, care pathways, patient populations, outcomes and improvement opportunities during the first quarter (Q1) of 2024 (January 1 to March 31, 2024).

## Methods

### Context

Both AGCHs operate within the Dutch healthcare system, which is based on three key principles: (1) universal access to healthcare with regulated competition; (2) solidarity through mandatory medical insurance for all residents; and (3) the provision of high-quality healthcare services [14]. Three healthcare laws apply to care for older people (see Supplementary File 1).

The AGCH model was developed between 2016 and 2018. The first AGCH (AGCH1) opened in mid-2018 within a skilled nursing facility and expanded from 12 to 23 beds by early 2024. The second AGCH (AGCH2) opened in late 2020 within an intermediate care ward at a general hospital, increasing its capacity from two to four beds in 2024. Both AGCHs were established as joint initiatives between a hospital, a nursing home organisation, and a health insurer. The partnering hospital of AGCH1 does not have a geriatric ward; that of AGCH2 does, but is located at a separate site approximately 70 km away. Both AGCH1 and AGCH2 were members of the AGCH learning network between 2021 and 2025, collaborating with seven other pilot sites to further develop the AGCH model. In 2024, AGCH care did not have a structural payment title but was temporarily financed through a daily tariff under an experimental payment title.

### Study design

Our study design was guided by the Medical Research Council (MRC) framework for complex interventions [15] and structured using the RE-AIM framework [16]. Reporting followed the STROBE [17] and COREQ [18] guidelines (see Supplementary File 2). The study protocol was submitted to the Amsterdam UMC ethics committee (file number 2025.0009), which waived formal approval. Written informed consent was obtained from all participants.

### Participants and research team

Both AGCHs were invited to participate. Recruitment started with online meetings with site managers, who then received an information letter and participated in the questionnaire, provided case vignettes, and invited focus group participants. Experts from the AGCH Learning Network were invited via email to evaluate the case vignettes. Table S3.1 in Supplementary File 3 lists all study participants.

### Data collection

Data collection (May 2024 – January 2025) included questionnaires (n = 2), documents (n = 10), case vignettes (n = 20), case vignette surveys (n = 7), and focus groups (n = 2). Table S3.2 in Supplementary File 3 links the RE-AIM domains to the data sources.

#### Questionnaires

Two site managers from each AGCH completed a questionnaire (see Supplementary File 4), providing information across four domains:

1. Context: setting, patient population, diagnostic and treatment resources, and full-time equivalent (FTE) staffing levels between January 1 and March 31, 2024;
2. Care delivery: extent of implementation of the core model elements between January 1 and March 31, 2024, assessed using a 5-point Likert scale;
3. Outcomes: aggregated outcomes on key performance indicators (KPIs) for patients admitted between January 1 and March 31 2024 (defined by the Dutch health insurers), as well as the percentage of patients requiring imaging outside the AGCH during admission (based on the hypothesis that AGCH2 would use hospital facilities more frequently due to its location within the hospital).
4. Patient characteristics of patients admitted between January 1 and March 31, 2024: age, sex, living situation, marital status, born in the Netherlands, Charlson comorbidity index [19], dementia, pholypharmacy, primary admission diagnosis, delirium.

#### Documents

We collected six documents describing care processes from AGCH1 and four document describing care processes from AGCH2. These documents were used to map the care pathways of both AGCHs, which were refined during the focus groups.

#### Case vignettes

We collected 10 case vignettes from each AGCH to explore referral appropriateness. The vignettes were provided by the coordinating physicians (a hospital geriatrician at AGCH1 and an ECP at AGCH2) and were based on real patients admitted to the two AGCHs. Anonymised information was collected using a structured case vignette format (see Supplementary File 5, Table S5.1), adapted from our previous AGCH case vignette study [20]. The coordinating physicians contributed five vignettes based on recently discharged patients, one vignette of a patient requiring evening, night, or weekend (ENW) care by a hospital geriatrician, ECP, nurse practitioner, or physician assistant, and four vignettes they considered representative of a typical AGCH patient.

#### Case vignette surveys

After collecting 10 case vignettes from each AGCH, we distributed case vignette surveys (see Supplementary File 5, Table S5.2). These surveys were adapted from our previous AGCH case vignette study [20] and were sent to seven independent experts not affiliated with AGCH1 or AGCH2 (see Table S3.1 in Supplementary File 3). In the survey, experts assessed five or six case vignettes and indicated whether they considered referral to the AGCH appropriate and, if not, which type of care they deemed more appropriate. Each case vignette (n = 20) was assessed by two experts.

#### Focus groups

At each AGCH, we conducted a focus group with participants (n = 4 and n = 8), including site managers, hospital geriatricians, ECPs, nurses, and physiotherapists; at AGCH2, a representative from the health insurer was also included (see Table S3.1 in Supplementary File 3). Discussions followed the Metro Mapping framework [21] and were structured around the phases of the AGCH care pathway: referral, admission, intake, treatment, discharge preparation, and discharge. Improvement opportunities were discussed across these phases, focusing on communication with patients and families, coordination among professionals, and contextual factors (see Supplementary File 7). The focus groups lasted 90–120 minutes, were recorded and transcribed verbatim.

### Data analysis

#### Qualitative anaysis

Thematic analysis was conducted in MAXQDA (version 2024) by LK and EK using both deductive and inductive approaches [22]. A priori codes were derived from the RE-AIM framework [16], the AGCH‘s core model elements, the AGCH’s admission criteria [20] and Brody et al.’s framework on challenges, solutions and scalability implications [23]. Themes were adapted, added, or removed if not supported by the data (see Supplementary File 8).

#### Quantitative analysis

Quantitative data on patient populations and KPI outcomes were collected and presented in aggregated form for each AGCH.

#### Member checks

Preliminary results were discussed with AGCH managers and shared by email with the coordinating physicians of each AGCH for member checking.

## Results

### Setting

AGCH1 is located in a large city and situated approximately two kilometres from its partnering university hospital. Patients are referred from the emergency department (ED) and acute admission units of the university hospital, as well as from the EDs of nearby top-clinical and general hospitals. After assessment by the on-call hospital geriatrician and consent from the patient or their representative, patients are transferred by ambulance. In Q1 2024, direct GP referral to AGCH1 was possible but rare. In- and exclusion criteria are shown in Table 1.

**Table 1:** Setting and admission criteria in the first quarter of 2024.


	AGCH1	AGCH2

**Location**	Standalone skilled nursing facility	Intermediate care ward within a general hospital

**Number of beds**	23	28

Nr. of AGCH beds	23	4

Nr. of GR beds	0	24

**Medical resources**		

Diagnostics	ECG, Early Sense beds, blood pressure monitor, urimeter, bladder scan, pulse oximeter, INR POCT meter, POCT glucose meter, daily laboratory testing and/or cultures (e.g., wound, urine, sputum, faeces) via external laboratory, radiological imaging (available once per week).	ECG, blood pressure monitor, urimeter, bladder scan, pulse oximeter, INR POCT meter, POCT glucose meter, daily laboratory testing and/or cultures (e.g., wound, urine, sputum, faeces) via external laboratory, radiological imaging (available Monday to Friday).

Medical treatment	Peripheral IV line, PICC line (IV medication administered via syringe pump, volumetric pump, or bolus), oxygen concentrators (up to 20 L/min per patient), portable oxygen cylinders, nebulization, indwelling urinary catheter, venipuncture, tube feeding, nasogastric tube, gravity drainage or PEG, stoma care, compression bandaging, specialized wound care, isolation with contact and airborne precautions, freedom-restricting interventions (e.g., bed rails, sensors).	Peripheral IV line, PICC line (IV medication administered via syringe pump, volumetric pump, or bolus), oxygen concentrators (up to 10 L/min per patient), portable oxygen cylinders, nebulization, VAC therapy, indwelling urinary catheter, venipuncture, tube feeding, nasogastric tube, gravity drainage or PEG, drain care, stoma care, compression bandaging, specialized wound care, suturing and suture removal, isolation with contact and airborne precautions, freedom-restricting interventions (e.g., bed rails, sensors).

Paramedical treatment	Daily physiotherapy from Monday to Saturday (with training room available in the building). Treatment by dietician and occupational therapist on request.	Daily physiotherapy from Monday to Friday (with training room available in the building). Treatment by dietician, occupational therapist, speech therapist, spiritual caregiver and social worker on request.

**Admission route**	Through the EDs and the acute admission units of general and academic hospitals.	Through the ED and the subacute geriatric unit of a general hospital.

**Inclusion criteria**	Patients aged 65 years or older (or clearly a geriatric patient, e.g., known to the geriatric outpatient clinic) Acute medical care demand: - Cardiology: congestive heart failure - Pulmonology: respiratory tract infection or pneumonia, COPD exacerbation - Neurology: neurological observation after, for example, a fall, delirium, or unknown cause. - Internal medicine: infections (such as urinary tract or soft tissue infections), poorly controlled diabetes, dehydration, electrolyte disturbance - Pain due to osteoporotic vertebral compression fracture, osteoarthritis, etc. One or more geriatric issues: Delirium, dementia, increased fall risk, reduced self-sufficiency	Patients aged 70 years or older Acute medical care demand: - Cardiology: mild form of congestive heart failure; - Pulmonology: upper respiratory tract infection combined with COPD exacerbation and/or congestive heart failure; - Neurology: no known inclusion criteria - Internal medicine: infections with or without delirium, dehydration with or without the first three criteria; - Pain due to osteoporotic vertebral compression fracture, osteoarthritis, etc.

**Exclusion criteria**	Hemodynamically unstable (expected need for ICU care), severe behavioral issues, recent TIA or stroke (due to absence of daily occupational and speech therapy).	Medically unstable, risk of wandering, severe behavioural issues.


AGCH = Acute Geriatric Community Hospital, GR = Geriatric Rehabilitation, ECG = Electrocardiogram, INR = International Normalized Ratio, POCT = Point-of-Care Testing, IV = Intravenous, PICC = Peripherally Inserted Central Catheter, VAC = Vacuum-Assisted Closure, PEG = Percutaneous Endoscopic Gastrostomy, ED = Emergency Department, COPD = Chronic Obstructive Pulmonary Disease, ICU = Intensive Care Unit.

AGCH2 is situated in a small city, housed within an intermediate care ward of a general hospital, run by a nursing care organisation. Patients are referred from the hospital’s ED and the subacute geriatric unit (SGU). During office hours, admission eligibility is jointly assessed by an ECP and a hospital geriatrician. Outside office hours, this assessment is conducted by a medical resident at the ED. Once consent is obtained, patients are admitted directly.

Medical resources available at both sites are presented in Table 1. AGCH1 partners with an institutional pharmacy that delivers medication daily and also maintains an in-house medication stock. Both AGCHs use Baxter medication rolls. AGCH2 collaborates with a 24/7 primary care pharmacy located within the hospital. In AGCH1, physiotherapy is available from Monday to Saturday, whereas in AGCH2 it is available from Monday to Friday.

### Staffing

AGCH1 employed 41.27 FTE care professionals for 23 AGCH beds, while AGCH2 employed 7.39 FTE for 4 beds (see Table 2). AGCH1 had a higher overall educational level among staff. At AGCH1, care is coordinated by a nurse practitioner, physician assistant, or medical resident, in collaboration with a hospital geriatrician and/or internist. At AGCH2, care is coordinated by an ECP or medical resident, with a hospital geriatrician acting as co-treating physician. Differences are also evident in the composition of the nursing team (see Table 2). In addition, AGCH2 employs an occupational therapist and dietician, whereas AGCH1 accesses these professionals on a consultative basis through partnering nursing home organisations.

**Table 2:** Staffing in the first quarter of 2024.


	AGCH1	AGCH2

Geriatrician or internist	6%	1%

Elderly care physician	0%	3%

Nurse specialist	7%	0%

Physician assistant	5%	1%

Medical resident	6%	11%

Registered nurse (bachelor’s degree)	17%	0%

Registered nurse (associate’s degree)	34%	32%

Nursing assistants	2%	12%

Healthcare assistants	9%	22%

Physical therapist	6%	8%

Dietician	0%	1%

Occupational therapist	0%	1%

Medical secretaries	3%	4%

Team supervisor	2%	3%

Manager	2%	1%

Total FTE deployment	41.27 for 23 AGCH beds	7.39 for 4 AGCH beds


AGCH = Acute Geriatric Community Hospital, FTE = full-time equivalent.

### Care pathway

#### Personalized, specialistic, acute geriatric care

At both sites, a Comprehensive Geriatric Assessment (CGA) is initiated in the ED/SGU by a hospital geriatrician to guide an individualised, multidisciplinary treatment plan. If not completed prior to admission, the CGA is finalised at the AGCH. At AGCH1, admission eligibility is assessed by the hospital geriatrician. At AGCH2, this assessment is carried out jointly by the hospital geriatrician and the ECP during office hours. Bed availability is verified via WhatsApp at AGCH1 and via email at AGCH2. Medication and treatment handovers are conducted by phone and secured email at both sites.

Upon arrival, nurses at both AGCHs welcome the patient, provide orientation, answer questions, and facilitate care agreement signing. The patient’s GP record is requested, and a medication review is performed. The hospital geriatrician (together with the ECP at AGCH2) visits the patient the same day or the following day in case of evening admission. A physiotherapist conducts a separate intake at both sites; at AGCH2, this is done in collaboration with an occupational therapist. Within 24 hours, an admission interview of 30–45 minutes is held with the patient and their caregiver(s) to discuss the treatment plan, prior functioning, discharge goals, and (if needed) advance care planning is initiated.

AGCH1 uses EarlySense sensors for continuous physiological monitoring. AGCH2 relies on clinical observation, supported when necessary by a Rapid Response Team and medical specialists. Nurses at both AGCHs receive in-house training in early warning sign recognition and clinical reasoning.

#### Integrated care focused on returning home

At AGCH1, rehabilitation goals are defined within two days after admission. At AGCH2, these goals are set during weekly multidisciplinary team (MDT) meetings. The hospital geriatrician does not attend MDTs at AGCH2 but visits the ward daily to discuss patients with the ECP, focusing primarily on somatic progress. At AGCH1, MDTs are convened mainly for patients with complex needs or discharge challenges, with an ECP also present. Informal communication and coordination within the interdisciplinary team is frequent at both sites. Consultations with other medical specialists are rare.

Discharge preparation starts after the initial recovery phase, which typically lasts 5–7 days. At AGCH1, nurses coordinate the discharge process. At AGCH2, this task is led by the ECP in collaboration with care coordinators, who also participate in MDT meetings. At both sites, personal handovers to GPs, physical therapists, pharmacists, and/or community nurses are arranged via telephone and/or email. Discharge medications are prescribed and supplied as needed.

#### Prevention in areas of vulnerability

Both AGCHs apply the Dutch Safety Management Program to screen for risks such as functional decline, falls, delirium, and malnutrition, usually in the ED or SGU, or otherwise upon arrival at the AGCH. A positive frailty screen triggers targeted preventive measures, including delirium protocols, behavioural support, nutritional interventions, and fall prevention strategies. Early mobilisation is standard practice.

At AGCH1, physiotherapists provide specialised rehabilitation therapy six days a week (Monday to Saturday). At AGCH2, daily therapy is provided from Monday to Friday, with routine involvement of an occupational therapist. Recovery is also encouraged through daily activities.

#### Appropriate environment close to home

At both sites, patients stay in single rooms with private shower and toilet facilities, designed to reduce stimuli and lower the risk of delirium. Ultra-low beds, which can be lowered to floor level, are available at both AGCHs. AGCH1 also has two bariatric beds. Both AGCHs offer a homelike environment that supports mobility and rehabilitation, including access to exercise areas. AGCH2 also has shared communal spaces for social interaction and peer support.

Visiting hours are open throughout the day at AGCH1. At AGCH2, visiting is allowed from 10:00–12:00, 13:30–17:00, and 18:30–21:00. At AGCH1, caregivers may join patients for meals in their rooms, whereas meals at AGCH2 are served in a communal dining area where caregiver access is not permitted. At both sites, an extra bed is available for family members or caregivers who wish to stay overnight.

### Patient population

#### Patient characteristics

Between January 1 and March 31, 2024, a total of 132 patients were admitted to AGCH1 and 29 to AGCH2 (see Table 3). Most patients lived independently prior to admission (62.1% at AGCH1; 51.7% at AGCH2). Both sites showed a high comorbidity burden (mean CCI: 5.2 in AGCH1 and 5.9 in AGCH2). Dementia was more prevalent (27.6% vs. 15.2%) in AGCH2, whereas polypharmacy was less common in AGCH2 (65.5% vs. 79.5%). Delirium at admission was present in 22.7% of patients at AGCH1 and 31.0% at AGCH2. Respiratory conditions were the most common reason for admission at AGCH1 (36.4%), whereas infections other than respiratory were most prevalent at AGCH2 (44.7%).

**Table 3:** Patient characteristics per AGCH in the first quarter of 2024.


	*AGCH1 Q1 2024* (n = 132)	*AGCH2 Q1 2024* (n = 29)

Age in years, mean (SD)*	82 (7.7)	83 (8.6)

Men, n (%)*	55 (41.7%)	9 (31.0%)

Living situation, n (%)		

Independent alone	82 (62.1%)	15 (51.7%)

Living with others (partner, children)	37 (28.0%)	9 (31.0%)

Nursing home/assisted living	10 (7.6%)	1 (3.4%)

Missing, n (%)	3 (2.3%)	4 (13.8%)

Marital status, n (%)		

Married or living together	45 (34.1%)	11 (37.9%)

Single or divorced	39 (29.5%)	1 (3.4%)

Widow or widower	46 (34.8%)	13 (44.8%)

Missing, n (%)	2 (1.5%)	4 (13.8%)

Born in the Netherlands, n (%)*	95 (72.0%)	26 (89.7%)

Charlson Comorbidity Index^†^, mean (SD)*	5.2 (1.8)	5.9 (2.6)

Dementia, n (%)	20 (15.2%)	8 (27.6%)

Polypharmacy^‡^, n (%)	105 (79.5%)	19 (65.5%)

Missing, n (%)	0 (0)	5 (17.2%)

Primary admission diagnosis, n (%)*		

Respiratory (including infections)	48 (36.4%)	8 (27.6%)

Other infections	29 (21.9%)	13 (44.7%)

Gastrointestinal	5 (3.8%)	1 (3.4%)

Cardiac	12 (9.1%)	3 (10.3%)

Electrolyte disturbance	8 (6.1%)	0 (0%)

Neurology	5 (3.8%)	2 (6.8%)

Other	25 (19.0%)	2 (6.8%)

Delirium at admission*	30 (22.7%)	9 (31.0%)


AGCH = Acute Geriatric Community Hospital, Q1 = first quarter, SD = standard deviation. *No missing variables. ^†^Ranging from 0 to 31, with a higher score indicating more severe comorbidity [17]. ^‡^Use of five or more medications at the same time.

#### Referral appropriateness

**Figure 2:**
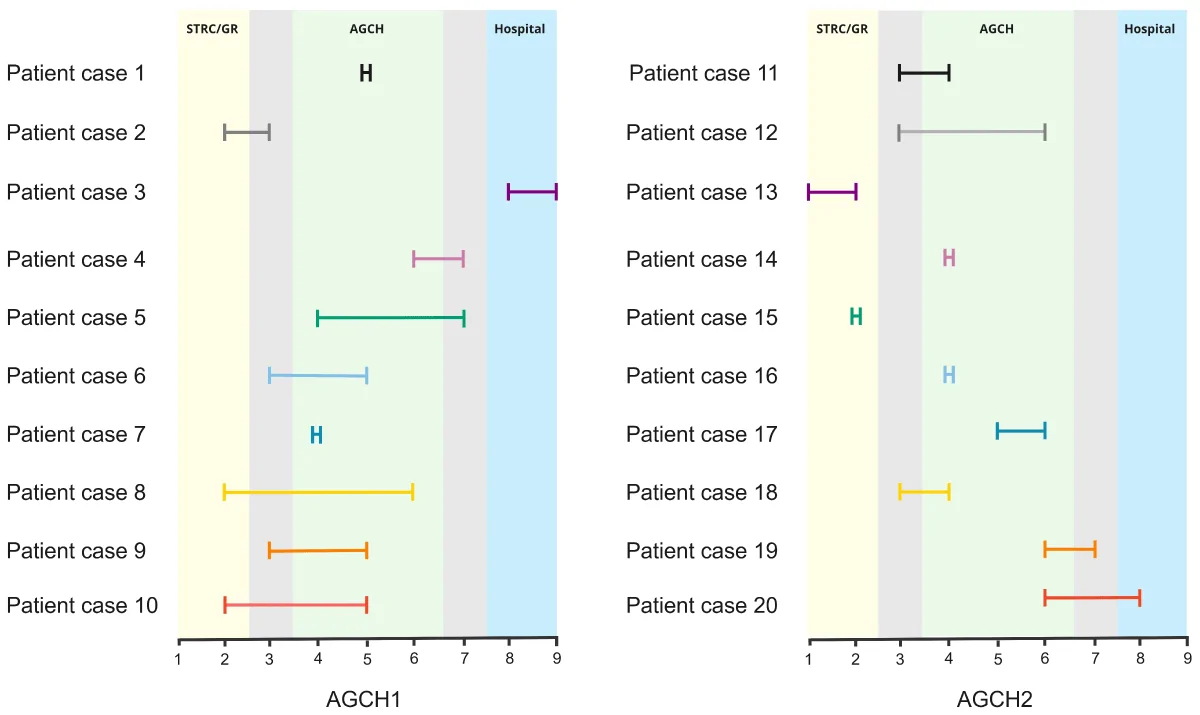
Referral appropriateness. AGCH = Acute Geriatric Community Hospital, STRC = Short-Term Residential Care, GR = Geriatric Rehabilitation. The yellow area represents referral decisions to STRC or GR: x = 1 indicates lower-complex primary care needs, and x = 2 indicates higher-complex primary care needs requiring further observation. The green area represents AGCH referral decisions: x = 4 indicates lower-complex AGCH care needs, x = 5 indicates medium-complex AGCH care needs, and x = 6 indicates higher-complex AGCH care needs. The blue area represents hospital referral decisions: x = 8 indicates lower-complex hospital care needs with location-specific requirements (e.g., closed ward or quarantine), while x = 9 indicates higher-complex hospital care needs. The grey areas represent referral decisions where the expert is uncertain whether the patient’s care needs align with STRC/GR or AGCH care (x = 3), or whether the patient’s care needs are more appropriate for AGCH or hospital care (x = 7).

Each AGCH site provided 10 anonymised case vignettes. These were assessed by two independent experts for referral appropriateness and, if deemed inappropriate, for the type of care considered more suitable. In Figure 2, whisker lines represent the range of both expert ratings. When both experts considered referral to the AGCH appropriate, the whisker line falls entirely within the green area (e.g. patient case 1 of AGCH1). If either end of the whisker line falls within the yellow area, an expert considered AGCH referral inappropriate and Short-Term Residential Care (STRC) or Geriatric Rehabilitation (GR) more appropriate. If either end falls within the blue area, the case was judged too complex for AGCH referral and hospital care was considered more appropriate. If either end of a whisker line fell within the grey area, one of the experts was uncertain whether the patient’s care needs aligned with STRC/GR or AGCH care (x = 3), or with AGCH or hospital care (x = 7).

For AGCH1, experts agreed that AGCH referral was appropriate for cases 1 and 7, whereas case 3 was considered too complex for the AGCH. Opinions diverged on the remaining cases. In cases 6 and 8, uncertainty centered on the need for intravenous (IV) versus oral antibiotics. Case 9 involved diagnostic uncertainty, while case 10 raised concerns due to psychiatric comorbidity and limited psychiatric support at the AGCH. In cases 2, 4 and 5, limited (clinical) information—such as cognitive status, renal function, delirium severity, support network and treatment preferences—complicated the assessment of referral appropriateness.

For AGCH2, the experts agreed on referral appropriateness for cases 14, 16, and 17, and that cases 13 and 15 did not require AGCH care. Opinions varied on the remaining five cases. For cases 11 and 12, the key issue was the route of antibiotic administration (IV or oral). In case 18, the presence of delirium raised questions about whether sufficient observation would also have been feasible at STRC or GR. Cases 19 and 20 raised concerns regarding medical instability and the feasibility of frequent monitoring and laboratory testing at the AGCH. Further case details are provided in Supplementary File 6.

### Outcomes

Table 4 presents KPI outcomes for AGCH1 (n = 132) and AGCH2 (n = 29) for admissions between January 1 and March 31, 2024.

**Table 4:** Key performance indicators in the first quarter of 2024.


	*AGCH1 Q1 2024 N = 132*	*AGCH2 Q1 2024 N = 29*

Length of AGCH admission*		

Average LOS (days)	12.5	9

Median LOS (days)	9	–

Discharge destination, n (%)		

Home, with or without home care, with or without LTC	90 (70.3%)	15 (51.7%)

STRC	8 (6.3%)	0 (0%)

GR	10 (7.8%)	3 (10.3%)

Nursing home or assisted living	7 (5.5%)	3 (10.3%)

Hospital (with or without ED visit)	4 (3.1%)	2 (6.9%)

Not applicable (e.g. patient deceased)	9 (7.0%)	2 (6.9%)

Missing	4 (3.0%)	4 (13.8%)

ED or hospital visits for imaging during AGCH admission, (%)^†^	2 (1.6%)	2 (6.9%)


AGCH = Acute Geriatric Community Hospital, LOS = length of stay, LTC = long-term care, STRC = Short-Term Residential Care, GR = Geriatric Rehabilitation, ED = Emergency Department, Q1 = first quarter. *Missing LOS data for four patients at AGCH1. No missing LOS data for AGCH2. ^†^Missing data on imaging for four patients at AGCH1 and no missing for AGCH2.

Mean length of stay (LOS) was shorter at AGCH2 (9.0 days) than at AGCH1 (12.5 days); median LOS was not available for AGCH2. A higher proportion of patients were discharged home from AGCH1 (70.3%) compared to AGCH2 (51.7%). No patients at AGCH2 were discharged to STRC, compared to 6.3% at AGCH1. Discharges to the hospital, nursing homes, and assisted living were more frequent at AGCH2. Two patients at each site required ED imaging during admission. Additionally, three patients at AGCH1 received bedside X-rays, a service not available at AGCH2. For both partnering hospitals in Q1 2024, the proportion of patients aged ≥70 and ≥80 presenting at the ED and directly referred from there to the AGCH was low (see Table 5).

**Table 5:** ED discharge destinations in the first quarter of 2024.


	*PARTNERING HOSPITAL Q1 2024*	*PARTNERING HOSPITAL Q1 2024*

Number of 70+ patients admitted to the ED	1238	997

Discharge destination of 70+ patients admitted to the ED, n (%)		

The hospital where the patient initially presented at the ED	292 (23.6%)	461 (46.2%)

Another hospital	136 (11.0%)	7 (0.7%)

Home with or without district care, with or without LTC	700 (56.5%)	489 (49.0%)

AGCH	37 (3.0%)	29 (2.9%)

STRC and GR	^¥^2 (0.2%)	8 (0.8%)

Not applicable (e.g. patient deceased)	64 (5.2%)	3 (10.3%)

Missing	7 (0.6%)	0 (0%)

Number of 80+ patients admitted to the ED	524	448

Discharge destination of 80+ patients admitted to the ED, n (%)		

The hospital where the patient initially presented at the ED	132 (25.2%)	220 (49.1%)

Another hospital	75 (14.3%)	3 (0.7%)

Home with or without district care, with or without LTC	258 (49.2%)	199 (44.4%)

AGCH	26 (5.0%)	16 (1.6%)

STRC and GR	0 (0%)	9 (0.9%)

Not applicable (e.g. patient deceased)	30 (5.7%)	1 (0.2%)

Missing	3 (0.6%)	0 (0%)


ED = Emergency Department, AGCH = Acute Geriatric Community Hospital, STRC = Short-Term Residential Care, GR = Geriatric Rehabilitation, Q1 = first quarter.

### Improvement opportunities

Focus group participants identified several key areas for improvement: appropriate referral, expectation management, discharge planning, IT integration and funding. At AGCH2, additional improvement opportunities were suggested for ENW care.

#### Appropriate referral

Focus group participants at both AGCHs reported inappropriate referrals. These mainly involved patients without a clear indication for specialist medical care, such as uncomplicated urinary tract infections requiring only brief IV fluids. Participants noted that such treatments could also be provided in STRC/GR settings. However, these patients were referred to the AGCH because admission to STRC/GR was not (24/7) available, combined with insufficient clinical information at referral.

To address this, focus group participants suggested improving ED referral decision-making tools, implementing real-time dashboards displaying intermediate care capacity, and enhancing transparency in communication between ED and AGCH staff. At AGCH2, participants proposed appointing a dedicated referral coordinator at the ED. At the other AGCH, focus group participants were hesitant to include AGCH bed availability in dashboards, fearing it might lead to inappropriate referrals when STRC, GR, or LTC beds are unavailable.

#### Managing expectations

Focus group participants at both AGCHs experienced challenges in aligning patient and caregiver expectations with the short-term nature of AGCH care. They identified inconsistent pre-admission communication, due to staff turnover and involvement of multiple professionals, as a barrier:

“*[Ambulance staff] sometimes say things like, ‘You can recover and regain your strength there.’ Well-intentioned, of course, but then I think ‘No, we’re here to treat first and then we’ll see if recovery is possible’*.”

– P4, focus group, 12/08/24

“*We have also had cases where families had already ended the tenancy on their parents’ home, saying ‘But the GP said it was fine and that you were going to sort it out*.’ ”

– P11, focus group, 09/09/24

Proposed improvements included consistent communication across care settings, teach-back methods, and standardised information materials for patients, caregivers, ED staff, ambulance personnel, and GPs.

#### Discharge planning

Discharge planning was challenging at both sites. In one location, nurses were responsible for both clinical care and discharge-related administrative tasks, such as completing nursing home applications, which contributed to delays:

*“You start it, but there’s no time to finish it in the afternoon, evening or at night. The next day, you pick it up again, fill in part of it, and before you know it, two or three days have passed. While ideally, it should be done within an hour on day one.”*

– P4, focus group, 12/08/24

In the other AGCH, care coordinators supported discharge, yet outflow remained delayed due to placement issues involving STRC, GR, nursing homes, home care, and hospices. Delays were also caused by pending home adaptations and overburdened informal caregivers. Participants proposed reclassifying patients as STRC, GR or ‘wrong bed’ once specialist care ended, to improve interpretation of AGCH LOS.

#### IT integration

Limited integration between hospital and nursing home electronic health record (EHR) systems hindered information exchange:

“*Nursing handovers are done by phone because we work with two different systems*.”

– P3, focus group, 12/08/24

This also delayed access to specialist consultations:

*“Sometimes we try [to request a specialist consult], but the process is slow because specialists are often unfamiliar with the AGCH. They then suggest a follow-up at the outpatient clinic weeks later instead. To make clear that the patient is hospitalised, we need to request a consult [through the EHR]. This isn’t always feasible.”*

– P5, focus group, 09/09/24

Participants stressed the need for interoperable IT systems and formalised agreements to ensure timely specialist involvement.

#### Funding

The temporary experimental payment title restricted long-term planning and scalability. Both sites emphasised the need for a structural payment title to ensure continuity and scalability.

#### ENW care

Focus group participants of AGCH2 shared specific challenges during ENW hours, including unclear medical responsibility, inappropriate referrals, and stagnating treatment plans over weekends. Proposed solutions included developing a formal AGCH care pathway at the ED to reduce reliance on verbal communication with rotating doctors; appointing a dedicated AGCH ECP instead of relying on external on-call services; and ensuring geriatric expertise at the ED during ENW hours.

## Discussion

This comparative process evaluation shows that the two AGCHs appeared to have largely similar care pathways, patient populations, and improvement opportunities in Q1 2024, but differed in context, staffing, and outcomes. At both AGCHs, patients presented with high multimorbidity, with respiratory conditions and infections being common diagnoses. Experts also reported variation in referral appropriateness. The mean LOS was shorter at AGCH2 (9.0 vs. 12.5 days), but a smaller proportion of patients were discharged home (51.7% vs. 70.3%). Improvement areas included referral, expectation management, discharge planning, IT integration, funding, and evening/night/weekend care.

These findings raise the question to what extent AGCH1 and AGCH2 implement the same complex intervention, given the observed differences in contextual and staffing characteristics (e.g. educational level of personnel and physiotherapy availability) alongside largely similar care pathways. We conclude that both AGCH1 and AGCH2 resemble the acute geriatric unit (AGU) model, such as the Subacute Care Unit (SCU) in Barcelona [9]. AGUs typically operate within a community hospital, intermediate care setting, or nursing home, and admit older patients with mild to moderate acute conditions when there is no major diagnostic uncertainty. AGCH1 is situated in a skilled nursing facility, while AGCH2 is located in a hospital ward with other intermediate care services (GR and STRC). Both AGCHs differ from Acute Care for Elders (ACE) units in the United States, which are designed to deliver comprehensive, age-friendly acute medical care fully within a hospital setting [24]. Unlike the AGCH, ACE units admit patients with diagnostic uncertainty or more complex diagnostic needs [9]. Nevertheless, key elements of ACE units and AGCHs overlap substantially [25].

This comparative process evaluation shows that both AGCHs in the Netherlands are characterised by close collaboration between the hospital and nursing home organisation, combining ‘cure’ and ‘care’ within a integrated care pathway close to home. However, implementing such a cross-sector model brings specific challenges. Several improvement areas identified in this study, such as appropriate referral, discharge planning, IT integration and funding, were also reported in the 2019–2021 process evaluation [26]. Our findings on expectation management also align with earlier work by Ribbink et al. (2021), which showed that patients often had limited understanding of what to expect from the AGCH [12]. This suggests that these challenges are peristent over time and across AGCH settings.

Compared to AGCH patients admitted in 2019–2020 as part of the prospective controlled observational study (n = 206), a larger proportion of patients in our Q1 2024 sample lived independently alone prior to admission (51.7%–62.1% vs. 48.5%), while fewer resided in nursing homes or assisted living facilities (3.4%–7.6% vs. 17.6%). The proportion of widowed patients was also lower (34.8%–44.8% vs. 46.6%). Average LOS increased at AGCH1 (12.5 vs. 9.8 days), and the proportion returning home slightly increased at AGCH1 (70.3% vs. 67.5%). Notably, patients in our study had a substantially higher mean Charlson Comorbidity Index compared with the AGCH 1 2019–2020 cohort (5.2–5.9 vs. 2.8) [11]. These findings, alongside longitudinal evidence of increasing multimorbidity among Dutch older adults [27,28], suggest that AGCHs will increasingly serve older adults with complex care needs who are ageing in place, potentially requiring further adaptation of the care model.

A key strength of this study is its mixed-methods design, guided by the MRC framework and structured along the RE-AIM dimensions. Methodological triangulation strengthened the depth and validity of our findings. Nonetheless, several limitations should be acknowledged. First, patient characteristics and KPI outcomes were collected and reported in aggregated form and covered only Q1 2024, which precluded meaningful statistical comparisons. Second, we did not include outcomes such as 30- or 90-day readmissions or changes in functioning over time, although these are relevant indicators for the AGCH. In consultation with health insurers, this study focused on the jointly defined KPIs. Third, data saturation was not reached. Not all key elements, such as ‘prevention in the areas of vulnerability’, were explored in depth during the focus groups. Scalability implications were also insufficiently addressed and were therefore excluded from the code tree (see Supplementary File 8) due to lack of supporting data. To enhance trustworthiness, we conducted member checks with AGCH teams and applied multiple triangulation techniques (methodological, data source, and investigator) [29].

This process evaluation has several implications. We recommend that providers regularly reassess the AGCH’s target population and referral procedures to ensure appropriate patient selection. To sustain and scale up the AGCH model, it is essential to maintain a continuous learning environment with sufficient flexibility to adapt to local contexts. Embedding the AGCH within an intermediate care ward may support more efficient use of capacity and enable smoother transitions to post-acute care. Moreover, successful implementation requires strong collaboration between the nursing home organisation and the hospital. Clear agreements should be in place regarding the division of coordinating roles and responsibilities among ECPs, nurse specialists, physician assistants, and hospital geriatricians. Given the substantial differences in the educational level of personnel between the two AGCHs, both settings may benefit from evaluating whether the current team composition provides the appropriate skill mix for the AGCH population and whether weekend availability of physiotherapy should be standard practice. A systematic review and meta-analysis by Hayes et al. (2024) suggests that early involvement of paramedical disciplines is essential for preventing functional decline and facilitating timely discharge in frail older populations [30]. In addition, health insurers and care providers should co-develop solutions for patients who are unlikely to return home within 14 days but may still benefit from AGCH care. And while AGCH1 has expanded to 23 beds, AGCH2 remains limited to four beds in 2024. We therefore recommend securing a dedicated location or ward for AGCH2 to enable higher-volume care and support more robust implementation and evaluation of its effectiveness. We also recommend scaling up the AGCH model in a controlled manner to additional providers to assess its effectiveness beyond the two sites included in this study.

In parallel, future research should focus on refining the model’s programme theory, strengthening patient involvement in evaluations, evaluating patient experiences in different AGCH settings, collecting longitudinal patient-level data to better assess AGCH health outcomes (such as hospital readmissions and ED visits) and conducting an economic evaluation. To support upscaling, the model’s generic functions should be clearly defined, distinguishing core from adaptable components (e.g. using the function–form distinction [31]). This will help balance fidelity and adaptability, minimise model drift, and facilitate successful implementation across diverse care settings. Finally, the AGCH should be studied within the broader context of regional Integrated Acute Care for Older people, rather than as an isolated intervention [32].

## Conclusions

The two AGCHs appear to have largely similar care pathways, patient populations, and improvement opportunities, but differed in context, staffing, and outcomes. To support sustainable implementation, we recommend continuous learning (to enable ongoing adaptation to the dynamic AGCH population), clear referral pathways and evaluation of the team’s skill mix. Scaling up the AGCH model requires effectiveness studies across diverse contexts and clearer role delineation among ECPs, nurse specialists, physician assistants, and hospital geriatricians. Defining core versus adaptable elements may help prevent model drift and facilitate implementation across different settings.

## Additional files

The additional files for this article can be found as follows:

10.5334/ijic.10214.s1 Supplementary File 1. Dutch healthcare system.

10.5334/ijic.10214.s2 Supplementary File 2. STROBE & COREQ checklists.

10.5334/ijic.10214.s3 Supplementary File 3. RE-AIM dimensions, data sources and respondents.

10.5334/ijic.10214.s4 Supplementary File 4. Questionnaire.

10.5334/ijic.10214.s5 Supplementary File 5. Case vignette format and survey.

10.5334/ijic.10214.s6 Supplementary File 6. Case vignette results.

10.5334/ijic.10214.s7 Supplementary File 7. Focus group interview guide.

10.5334/ijic.10214.s8 Supplementary File 8. Code tree.
