# Supplementary material for: Two Dutch Examples of Acute Geriatric Community Hospitals for Older Adults: A Comparative Process Evaluation
Source: Int J Integr Care. 2026 Sep 8;26(3):18. doi: 10.5334/ijic.10214 (PMC13564377; doi:10.5334/ijic.10214)
Supplement: Supplementary file 1 [file ijic-26-3-10214-s1.pdf]

SUPPLEMENTARY FILE 1: DUTCH CONTEXT

The Dutch healthcare system

The Dutch healthcare system is broadly based on three principles: universal access to care, solidarity through mandatory medical insurance (which is provided to all), and high-quality healthcare services [1]. The system is shaped by several historical trends, changes and social conditions. The reforms in 2006 changed the role of the stakeholders and actors in the healthcare sector, while the 2015 reform was targeted towards containing costs related to long-term care [2].

Public and private insurance are merged through a universal social health insurance approach. All residents are required to purchase statutory health insurance from private insurers, who are required to accept all applicants. All insurers are mandated to operate as nonprofits. Financing is primarily public, through premiums, tax revenues, and government grants. The national government is responsible for setting health care priorities and monitoring access, quality and costs. Standard benefits include hospital, physician, home nursing, and mental health care, as well as prescription drugs. Citizens pay premiums, annual deductibles, and coinsurance or copayments on select services and drugs [3].

Three basic health care-related acts applying to care for older people

Three of the four basic health care-related acts that govern the healthcare system apply to care for older people in the Netherlands:

1. The Health Insurance Act (in Dutch: Zorgverzekeringswet) provides short-term medical care, such as general practitioner services, hospital care, prescription drugs, and mental healthcare [4]. This act accounts for the largest amount of the healthcare budget. Private insurance companies play a key role in implementing the act in a system based on “regulated competition” [1]. They must reimburse the standard benefits which are insured under the mandatory benefit package. The national government determines this statutory benefit package. Short-term medical care financed through the Health Insurance Act is provided by private entities and profits may not be distributed to the stakeholders [3].
   - ➔ Care for older people provided through the statutory benefit package includes for example hospital care, district nursing, temporary medical care for specific patient groups living at home and intermediate care (Geriatric Revalidation and Short-Term Residential Care).
2. The Long-Term Care Act (in Dutch: Wet langdurige zorg) provides long-term care for vulnerable groups such as older people with frailty, people with chronic illnesses, people with severe mental or physical disabilities. Those requiring permanent supervision and 24-hour care are entitled services under this act [1]. A special assessment center (the CIZ) determines whether a person is eligible [4]. Long-term care is covered through a state-controlled mandatory insurance and administered by regional long-term care administrators at the behest of the central government. Care is provided by private, nonprofit organizations [1].
   - ➔ The Long-Term Care Act applies to nursing at home or in a nursing home. Older people can obtain care through a contracted long-term care provider, but can also buy their own care at home through a personal budget.
3. The Social Support Act (in Dutch: Wet maatschappelijke ondersteuning) provides care to help people live independently at home and participate in society for as long as possible [1]. Social support services are financed through the municipal fund which is supplied to the (roughly 350) municipalities by the central government. The municipalities have a great deal of freedom in how they spend these funds in order to meet the requirement of the Social Support Act [3].
   - ➔ The Social Support Act provides general support to older people such as domestic help, house adjustments and transport. They can obtain care through a contracted social care provider, but can also buy their own social care through a personal budget.

References:

1. **Ministry of Health, Welfare and Sport.** Healthcare in the Netherlands. https://english.zorginstituutnederland.nl/ documents/2016/01/31/healthcare-in-the-netherlands (accessed 18 July 2025).
2. **Kroneman M, Boerma W, van den Berg M, Groenewegen P, de Jong J, van Ginneken E.** Netherlands: Health System Review. *Health systems in transition*. 2016;18(2):1-240. https://iris.who.int/bitstream/handle/10665/330244/HiT-18-2-2016-eng.pdf?
3. **Wammes J, Jeurissen P, Westert G, Tanke M.** The Dutch health care system. International profiles of health care systems. 2020;137(6). https://www.commonwealthfund.org/sites/default/files/2020-12/International_Profiles_of_Health_Care_Systems_Dec2020.pdf

4. **Varkevisser M, Schut E, Franken F, van der Geest S**. Sustainability and resilience in the Dutch Health System. *PHSSR* https://pure.eur.nl/en/publications/sustainability-and-resilience-in-the-dutch-health-system

Bed-based intermediate care models in the Netherlands

Table S1. Bed-based intermediate care models in the Netherlands

Bed-based intermediate care for frail older adults in the Netherlands		
**Geriatric revalidation**	**Short-term residential stay**	**Acute Geriatric Community Hospital**
Definition: Post-acute multidisciplinary (para)medic care for older and frail patients, including those with pre-existing functional decline or specific care needs.	*Definition:* Medical care for older adults with general health problems that do not require specialist care nor geriatric rehabilitation, but whose treatment and care needs cannot be met at home.	*Definition:* (Sub)acute specialized geriatric medical care for older patients with frailty.
Goal: To optimize functional capacities and support societal participation despite impairments, so that frail and/or older individuals can return home and live independently in the community.	*Goal:* Recovery so that older adults can return home and live independently in the community.	*Goal:* To provide medical specialist care for frail older adults in an adapted environment, so they can return home, live independently in the community and hospital (re)admissions are prevented.
Admission route:<br>Admission from hospital, ED, or home.<br>Referral by a medical specialist or ECP using a comprehensive geriatric assessment.	*Admission route:*<br>Admission from home, ED, or hospital.<br>Referral by GP or medical specialist.	*Admission route:*<br>Admission from ED.<br>Referral by a medical specialist.
Admission criteria:<br>(i). Medical stability<br>(ii). Multidisciplinary rehabilitation needs<br>(iii). Frailty and/or multimorbidity<br>(iv). Motivation/preference to undergo rehabilitation treatment<br>(v). A cognitive and physical status that allows participation in geriatric rehabilitation.<br><br>Targeted diagnoses: (1) stroke, (2) elective orthopedics, (3) trauma surgery (e.g., hip fractures), (4) amputations, and (5) other disorders (neurodegenerative diseases, oncological diseases, COPD, cardiac failure, internal- and multi-system failure).	*Admission criteria:*<br>No guidelines or targeted patient groups.	*Admission criteria (before 2023)*:<br>(i). Older patient with an acute medical problem that requires hospitalization, such as pneumonia or exacerbation of chronic conditions such as heart failure<br>(ii). Geriatric conditions (e.g. delirium, cognitive/functional impairment, falls)<br>(iii). Hemodynamic stability<br>(iv). No complex diagnostic testing needed such as CT or MRI scans during admission<br>(v). Return to previous living situation expected in 14 days
Staffing: A multidisciplinary team with special training in rehabilitation consisting of the elderly care physician, nurses, carers, physical therapists, psychologists, dieticians, social workers, and behavioral scientists.	*Staffing:* A multidisciplinary team consisting of the elderly care physician (or general practitioner), nurses, carers, physical therapists, and other paramedics if needed.	*Staffing:* An interdisciplinary team of healthcare professionals with geriatric expertise, including a geriatrician and/or medical specialist(s), elderly care physician, nurses, physical therapists, and other paramedics if needed.
Coordinating practitioner: The elderly care physician, nurse practitioner, or physician assistant.	*Coordinating practitioner:* The elderly care physician (high-complex STRC), general practitioner (low-complex STRC), nurse practitioner, or physician assistant.	*Coordinating practitioner:* The geriatrician, elderly care physician, nurse practitioner, or physician assistant.
Treatment:<br>A multidisciplinary set of evaluative, diagnostic and therapeutic interventions that are adapted to the rehabilitation needs of the frail elderly individual.<br><br>Palliative care cannot be provided in GR settings.	*Treatment:*<br>Three different STRC care paths exist:<br>- STRC low complex provides regular care for patients who are not in need for specific paramedic treatment, but temporarily need more care than homecare can provide.<br>- STRC high complex provides not only increased care, but also (multidisciplinary) treatment or rehabilitation in a slower pace than geriatric revalidation.<br>- STRC palliative care provides care for patients in the last 3 months of their life.	*Treatment:*<br>The four AGCH care components are:<br>(1) low-complex acute specialized geriatric care is safely provided<br>(2) care is patient-centred and focused on rehabilitation and return home (e.g. CGA, ACP, early rehabilitation, function focused care, caregivers involved during treatment (decisions))<br>(3) integrated care: transmural and close to home (e.g. comprehensive discharge planning (caregiver involved), warm handover to the GP and district nurse)<br>(4) fitting environment to prevent delirium and functional decline (e.g. rooming-in, noise reduction, management of delirium-inducing drugs).<br><br>Palliative care can be provided in AGCH settings.
Funding: Treatment, therapy and ADL care is funded according to a Diagnosis Treatment Combination (DTC).<br><br>--> For patients with a LTC indication, a LTC financing label ZZP 9b can be used.	*Funding:* Treatment, therapy and ADL care is funded according to a daily tariff, for a maximum of 6 months:<br>- STRC low complex: GP and paramedic treatment payment according to regular tariffs (as if patient would be home).<br>- STRC high complex: up to 1,5 hour treatment per week by ECP/paramedics.<br>- STRC hospice care: up to 3 hours treatment per week by GP/ECP/paramedics is funded.<br>--> For patients with a LTC indication, the LTC crisis financing labels (somatic or psychogeriatric) can be used.	*Funding:* Treatment, therapy and ADL care is funded according to a daily tariff, which is currently (2023) provided through an experimental financing structure.<br><br>--> For patients with a LTC indication no separate financing label has to be used under the experimental financing structure.

Abbreviations: ACP = Advance Care Planning; AGCH = Acute Geriatric Community Hospital; CGA = comprehensive geriatric assessment, COPD = chronic obstructive pulmonary disorder; CT = computed tomography; ED = emergency department; LTC = long-term care, MRI = magnetic resonance imaging; STRC = Short-Term Residential Care.
