# Supplementary material for: Two Dutch Examples of Acute Geriatric Community Hospitals for Older Adults: A Comparative Process Evaluation
Source: Int J Integr Care. 2026 Sep 8;26(3):18. doi: 10.5334/ijic.10214 (PMC13564377; doi:10.5334/ijic.10214)
Supplement: Supplementary file 3 [file ijic-26-3-10214-s3.pdf]

SUPPLEMENTARY FILE 3: RE-AIM DIMENSIONS, DATA SOURCES, OUTCOME MEASURES AND RESPONDENTS

Table S3.1. Study participants

Participant	Function	Site	Component of data collection
1	AGCH manager	AGCH1	Questionnaire
2	AGCH manager	AGCH1	Questionnaire
3	GR manager	AGCH2	Questionnaire
4	AGCH project manager	AGCH2	Questionnaire
5	Hospital geriatrician	AGCH1	Case vignette collection
6	Elderly care physician	AGCH2	Case vignette collection
7	Registered nurse (bachelor’s degree)	AGCH1	Focus group
8	Hospital geriatrician	AGCH1	Focus group
9	Interim manager	AGCH1	Focus group
10	Registered nurse (bachelor’s degree)	AGCH1	Focus group
11	Elderly care physician	AGCH2	Focus group
12	AGCH project manager	AGCH2	Focus group
13	Physical therapist	AGCH2	Focus group
14	AGCH project manager	AGCH2	Focus group
15	Health insurer	AGCH2	Focus group
16	GR manager	AGCH2	Focus group
17	Registered nurse (associate’s degree)	AGCH2	Focus group
18	Registered nurse (associate’s degree)	AGCH2	Focus group
19	Elderly care physician	Pilot site 1	Case vignette survey
20	Physician specialized in internal medicine and geriatrics	Pilot site 2	Case vignette survey
21	Elderly care physician	Pilot site 2	Case vignette survey
22	Elderly care physician	Pilot site 3	Case vignette survey
23	Physician specialized in internal medicine and geriatrics	Pilot site 4	Case vignette survey
24	Physician specialized in internal medicine and geriatrics	Pilot site 5	Case vignette survey
25	Elderly care physician	Pilot site 6	Case vignette survey

RE-AIM = , AGCH = Acute Geriatric Community Hospital, ED = Emergency Department, LOS = Length Of Stay, LTC= Long-term Care, STRC = Short-Term Residential Care, GR= Geriatric Rehabilitation.

Table S3.2. RE-AIM dimensions, data sources and outcome measures

RE-AIM dimension	Definition	Data sources	Outcome measures
**Reach**	The absolute number and representativeness of patients attending the AGCH sites.	Questionnaires<br>Case vignettes<br>Case vignette surveys	Patient characteristics<br>Referral appropriateness
**Adoption**	The extent to which stakeholders in the care pathway are willing to adopt the AGCH model and reasons for adoption or non-adoption.	Questionnaires<br>Documents<br>Focus group	Setting<br>Staffing<br>Improvement opportunities
**Implementation**	The implementation fidelity to the elements of the AGCH model, including consistency of delivery as intended and situational adaptations made.	Questionnaires<br>Focus groups	Care pathway
**Effectiveness**	The impact of AGCH on important outcomes.	Questionnaires	Outcomes:<br>1. Average and median length of stay<br>2. Percentage of AGCH patients discharged to home<br>3. Percentage of AGCH patients referred to the ED/hospital for imaging<br>4. Percentage of 70+ ED patients discharged to the AGCH<br>5. Percentage of 80+ ED patients discharged to the AGCH
**Maintainance**	The extent to which the AGCH model becomes institutionalized and part of the routine practices and policies.	Focus groups	Improvement opportunities

AGCH = Acute Geriatric Community Hospital, ED = Emergency Department, LOS = Length Of Stay, LTC= Long-term Care, STRC = Short-Term Residential Care, GR= Geriatric Rehabilitation.
