# Supplementary material for: Two Dutch Examples of Acute Geriatric Community Hospitals for Older Adults: A Comparative Process Evaluation
Source: Int J Integr Care. 2026 Sep 8;26(3):18. doi: 10.5334/ijic.10214 (PMC13564377; doi:10.5334/ijic.10214)
Supplement: Supplementary file 4 [file ijic-26-3-10214-s4.pdf]

SUPPLEMENTARY FILE 4: QUESTIONNAIRE

Demographic details of participant

1. What is your current position?
2. Since when have you held this position?
3. Since when have you been involved in (implementing) the AGCH in your region?
4. How long have you been working in healthcare?

AGCH context

5. When was the AGCH opened?
6. How many beds are currently open in the AGCH? (reference date: 1 May 2024)
7. Are there plans to increase or decrease the number of beds? If yes, when and to what number?
8. What is the location of the AGCH in relation to the partnering hospital?
   ☐The AGCH is located in a general hospital
   ☐The AGCH is located in a nursing home
   ☐The AGCH is located at a geriatric rehabilitation site
   ☐Other, namely:
9. What is the setting of the AGCH?
10. How many intermediate care beds (e.g. STRC, GR, LTC crisis) are there at the same location as the AGCH? (reference date: 1 May 2024)

Patient population

11. When does the AGCH admit patients? Only weekdays during the day, or also evenings and weekends?
12. What inclusion and exclusion criteria are used by your AGCH?
13. Who is involved in the decision to admit a patient to the AGCH?
14. How would you assess the complexity of the patients admitted to the community hospital? (see guideline referral decision-making to the AGCH) (Reference period: Q1 2024)
    Please draw a frame to indicate the spectrum of patients admitted in Q1 2024.

Diagnostic and treatment resources

15. What diagnostic options are available for patients in the AGCH, within the building where the AGCH is located?
    ☐Electrocardiogram (ECG)
    ☐Urimeter
    ☐Bladder scan
    ☐Pulse oximeter
    ☐POCT equipment, namely the following:
    ☐Laboratory tests. If so, how often and (via) where is this possible?
    ☐Echo-pocus
    ☐X-ray. If so, how often and (via) where is this possible?
    ☐Other, namely:
16. What treatment interventions are available for patients in the AGCH, within the building where the AGCH is located?
    ☐Intravenous medication, via a:
      ☐Peripheral line
      ☐PICC line
      ☐Central venous catheter (CVC)
      ☐Other, namely:
    ☐Oxygen. If so, the maximum litres per minute is:
    ☐Nebulisation
    ☐VAC therapy
    ☐Tracheostomy cannula
    ☐(Blood) transfusion
    ☐Indwelling urinary catheter (CAD)
    ☐Other, namely:

Staffing

17. What does the staffing structure (in FTEs) of your AGCH look like?

Function	FTE
Geriatrician or internist	
Elderly care physician	

Nurse specialist
Physician assistant
Medical resident
Registered nurse (bachelor’s degree)
Registered nurse (associate’s degree)
Nursing assistants
Healthcare assistants
Physical therapist
Dietician
Occupational therapist
Medical secretaries
Manager
**Total FTE deployment**

18. Are external disciplines ever consulted in your AGCH?

Core model elements

19. Estimating to what extent the community hospital meets the core components
(*Tick what applies – Always / Often / Sometimes / Rarely / Never / Not applicable*)

Person-centered specialist acute geriatric care:
a. Is a Comprehensive Geriatric Assessment (CGA) performed at intake?
b. Is proactive care planning carried out during admission?
c. Are (recovery) goals discussed beforehand with the patient and family?
d. Are treatment preferences and treatment limitations discussed?
e. Is a medical specialist involved in treatment?
f. Are multidisciplinary team meetings held?
g. Is continuous cardiac and respiratory monitoring possible?
h. Is there a tour and/or admission interview with the patient and family and/or informal caregiver(s)?

Prevention in domains of frailty:
i. Is there early rehabilitation and care focused on functioning to maintain/restore the patient's independence?
j. Is the risk of falls taken into account?
k. Is the risk of delirium taken into account?
l. Is the risk of malnutrition taken into account?
m. Is the risk of pressure ulcers taken into account?

A suitable environment close to home:
n. Does the patient stay in a single room?
o. Is the room equipped with necessary modifications (e.g. shower chair, toilet rails)?
p. Is the room homely in design?
q. Are corridors and halls adapted (e.g. handrails) and free of obstructions?
r. Can the patient go to a common room for social contact?
s. Can family and/or informal caregiver(s) join for meals?
t. Is rooming-in possible for family and/or informal caregiver(s)?
u. Are visiting hours more flexible than in a hospital?
v. Is there a recovery-oriented environment?
w. Are noise and stimuli minimised as much as possible?
x. Is “home” nearby for the patient (<30 min travel time)?

Integrated care for return home:
y. Does the team consist of professionals with backgrounds in both ‘care’ and ‘cure’?
z. Does the team have insight into the patient’s care network?
aa. Are professionals from this care network (if needed) involved during admission and/or treatment?
bb. Are two multidisciplinary meetings (MDT) held per patient?
cc. Are (provisional) discharge date and destination discussed during these MDTs?
dd. Is there personal contact with the GP/nursing home doctor to discuss the planned handover?
ee. Is there personal contact with the community nurse and/or physiotherapist to discuss the planned handover (if applicable)?
ff. Is a conversation with family and/or informal caregiver(s) planned at discharge?
gg. Is there coordination with family and/or informal caregivers at other moments (than admission, discharge, MDTs)?
hh. Is a discharge letter sent to the GP within 24 hours?
ii. Is medication provided for the first few days at home (if needed)?

Key Performance Indicators

20. How many patients were admitted to the AGCH between January 1 and March 31, 2024 (Q1)?
21. How many of these patients (Q1 2024) were referred back to the ED during admission to the AGCH, but were not admitted to the hospital? *(In numbers, not percentages)*
22. How many of these patients (Q1 2024) were (re)referred and admitted to the hospital during their stay in the AGCH? (In numbers, not percentages; corresponds to total of q24e +q 24g)
23. How many of these patients (Q1 2024) were discharged to: *(In numbers, not percentages)*
    a. Home (with/without home care and with/without long-term care (LTC) indication)
    b. STRC
    c. GR
    d. Nursing home admission (LTC)
    e. ED with admission to hospital
    f. ED without admission to hospital, but return to AGCH
    g. Hospital admission (without ED)
    h. Died in the AGCH
    i. Other, namely:
24. What was the average and median length of stay of the patients admitted in Q1 2024? (Excluding deceased and referred-back patients – i.e. exclude patients of q24e, f, g, h)
    a. Average length of stay in days:
    b. Median length of stay in days:
25. How many patients aged 70 or older were admitted to the ED of your partnering hospital in Q1 2024? *(In numbers, not percentages)*
26. How many of these ED patients aged 70+ were discharged to the following destinations in Q1 2024? *(In numbers, not percentages)*
    a. Hospital admission
    b. Home (with/without home care and with/without long-term care (LTC) indication)
    c. STRC
    d. GR
    e. Nursing home admission (LTC)
    f. Died in the ED
    g. Other, namely:
27. How many patients aged 80 or older were admitted to the ED of your partnering hospital in Q1 2024? *(In numbers, not percentages)*
28. How many of these ED patients aged 80+ were discharged to the following destinations in Q1 2024? (In numbers, not percentages)
    a. Hospital admission
    b. Home (with/without home care and with/without long-term care (LTC) indication)
    c. STRC
    d. GR
    e. Nursing home admission (LTC)
    f. Died in the ED
    g. Other, namely:

Patient characteristics (first admission date between January 1 and March 31 2024)

1. Age in years at admission
    a. Age in years, mean:
    b. Age in years, standard deviation:
    c. Missing, number of patients:
2. Sex
    a. Men, numbers of patients:
    b. Women, number of patients:
    c. Another or non-binary, number of patients:
    d. Missing, number of patients:
3. Living situation prior to admission
    a. Independent alone, number of patients:
    b. Living with others (partner, children) , number of patients:
    c. Nursing home or assisted living, number of patients:
    d. Missing, number of patients:

4. Marital status
    a. Married or living together, number of patients:
    b. Single or divorced, number of patients:
    c. Widow or widower, number of patients:
    d. Missing, number of patients:
5. Born in the Netherlands
    a. Yes, number of patients:
    b. No, number of patients:
    c. Missing, number of patients:
6. Charlson Comorbidity Index (CCI)*
    a. CCI, mean:
    b. CCI, standard deviation:
    c. Missing, number of patients:
7. Dementia
    a. Yes, number of patients:
    b. No, number of patients:
    c. Missing, number of patients:
8. Polypharmacy
    a. Yes, number of patients:
    b. No, number of patients:
    c. Missing, number of patients:
9. Primary admission diagnosis
    a. Respiratory (including infections), number of patients:
    b. Other infections, number of patients:
    c. Gastrointestinal, number of patients:
    d. Cardiac, number of patients:
    e. Electrolyte disturbance, number of patients:
    f. Neurology, number of patients:
    g. Other, number of patients:
    h. Missing, number of patients:
10. Delirium at admission
    a. Yes, number of patients:
    b. No, number of patients:
    c. Missing, number of patients:

* *Ranging from 0 to 31, with a higher score indicating more severe comorbidity.*

** *Use of five or more medications at the same time.*
