# Supplementary material for: Two Dutch Examples of Acute Geriatric Community Hospitals for Older Adults: A Comparative Process Evaluation
Source: Int J Integr Care. 2026 Sep 8;26(3):18. doi: 10.5334/ijic.10214 (PMC13564377; doi:10.5334/ijic.10214)
Supplement: Supplementary file 5 [file ijic-26-3-10214-s5.pdf]

SUPPLEMENTARY FILE 5: CASE VIGNETTE FORMAT AND SURVEY

Table S5.1. Case vignette format

**General Patient Information**	
Gender	…
Age	…
**Information about admission**	
Date of admission	…
Admission time (morning, afternoon, evening)	…
Submitted to the ED by	…
Submitted to the ED with the following problems(s)	…
**Patient history and background**	
Somatic	Differential diagnosis (based on, for example, lab results and scans): …<br>Medical history: …<br>List of current medications: …<br>Medication changes: …<br>Nutritional status: …
Psychological	Delirium: …<br>Cognitive status: …<br>Mood: …
Functional	Mobility: …<br>ADL: …<br>iADL: …
Social	Support system: …<br>Coping: …
Wishes with respect to treatment (advance care planning), and/or treatment limitations based on medical grounds	…
Diagnoses at arrival in the AGCH site	...
Medical stability	…
**Treatment in the AGCH site**	
Treatment plan	…
Social Functioning and Self-Management	Presence of an informal caregiver: …<br>Living situation: …<br>Health literacy: …<br>Coping mechanism: …
Estimated length of stay	… days
Intensity monitoring and treatment	Frequency of daily monitoring: …<br>Nighttime monitoring required: …
Deterioration risks	Fall incidents: …<br>Delirium: …<br>Malnutrition: …<br>Functional decline: …<br>Limitations in iADL/ADL: …<br>Bed sores: …<br>Complications: …<br>Acute deterioration: …
**Additional information patient cases**	
Estimated case complexity at the moment of admission	…
Which elements or combination of elements determine the complexity of a patient case? (Please mark all that apply)	Somatic (physical health)<br>Cognitive/Mental health<br>Social factors<br>Functional status
Was the treatment plan deviated from or adjusted during the patient's stay in the AGCH site?	…
Estimated case complexity during treatment	…
Medical specialist care provided (e.g., diagnostic: lab tests, ECG, POCUS, radiology; and therapeutic: close monitoring of vital signs, medication adjustments, etc.)	…
Through which pathway was the clinical geriatrician/elderly care internist involved? (Please mark all that apply)	Physical assessment<br>Telephone consultation<br>Multidisciplinary meeting<br>Other: _____________
Were other medical specialists involved in care? If so, which ones and why?	…
Through which route were other medical specialists involved? (Please mark all that apply)	Physical assessment<br>Telephone consultation<br>Multidisciplinary meeting<br>Other: _____________
Outpatient follow-up appointments	…

Table S5.2. Case vignette survey

**Fictional name**

1.	Under which fictional name is the patient case referred to?	…
**Complexity at time of AGCH admission**		
2.	How would you assess the complexity of the case at the time of admission, based on the spectrum of post-acute care in the figure below? (see ‘Guidance referral decision-making AGCH’) (Only consider information available at the time of admission, not information about the course during admission.)	☐Mango tree<br>☐Fig tree<br>☐Persimmon tree<br>☐Medlar tree<br>☐Cherry tree<br>☐Pear tree<br>☐Nectarine tree<br>☐Mandarin tree<br>☐Durian tree
	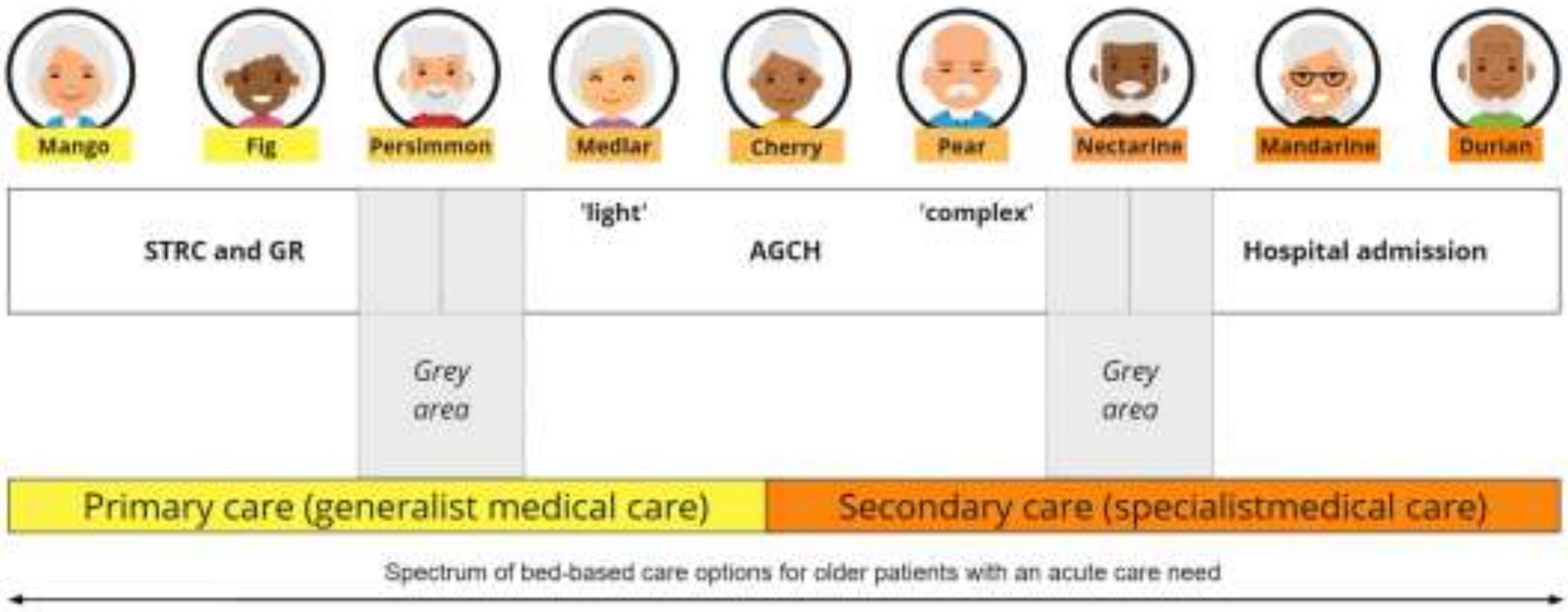	
**Complexity during AGCH admission**		
3.	Is information available for this patient case about the course of complexity) during admission at the AGCH?	☐Yes<br>☐No
4.	How would you assess the complexity of the patient case during admission at the AGCH? (see ‘Guidance referral decision-making AGCH’)	☐Mango tree<br>☐Fig tree<br>☐Persimmon tree<br>☐Medlar tree<br>☐Cherry tree<br>☐Pear tree<br>☐Nectarine tree<br>☐Mandarin tree<br>☐Durian tree
	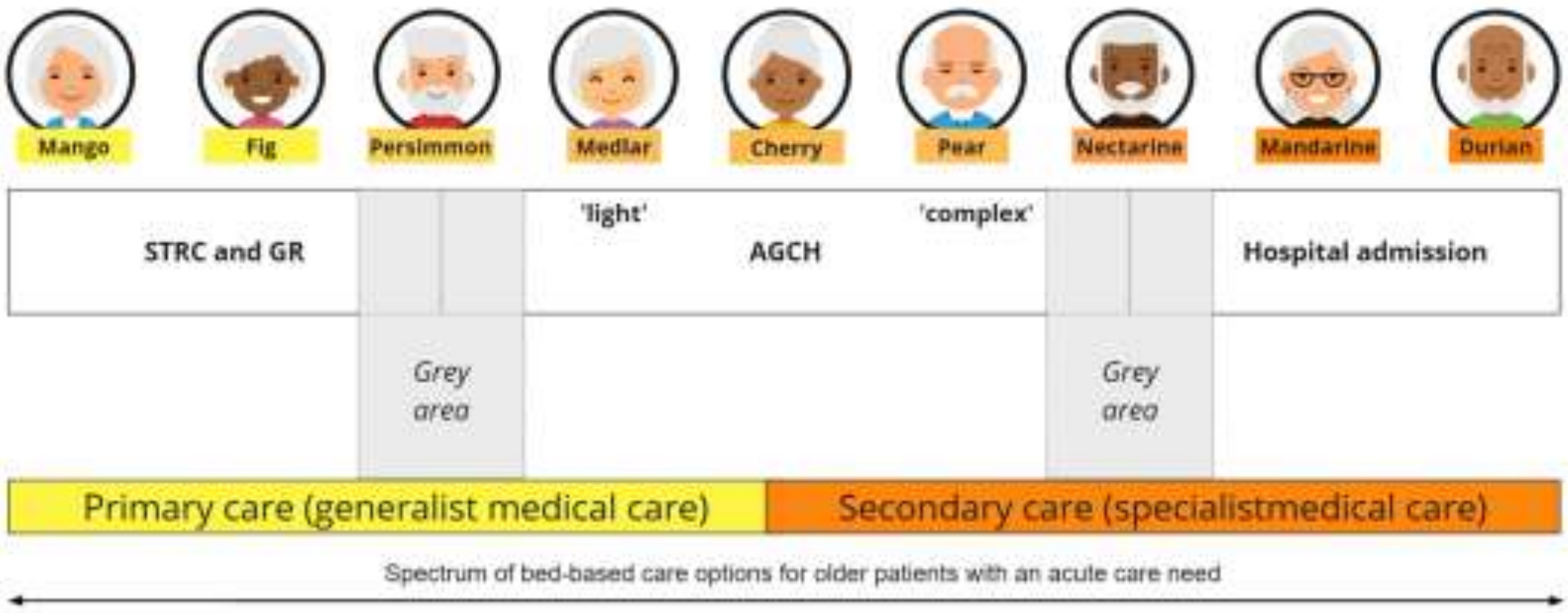	
**Complexity elements, considerations and/or dilemmas**		
5.	Which (combination of) elements in the patient case determine its complexity?	☐Somatic<br>☐Cognitive/mental<br>☐Social<br>☐Functional<br>☐Other
6.	Are there any considerations and/or dilemmas you would like to share with us?	…
7.	Was any information missing during referral decision-making?	☐Yes<br>☐No
8.	What information was missing during referral decision-making?	…
