# Supplementary material for: Two Dutch Examples of Acute Geriatric Community Hospitals for Older Adults: A Comparative Process Evaluation
Source: Int J Integr Care. 2026 Sep 8;26(3):18. doi: 10.5334/ijic.10214 (PMC13564377; doi:10.5334/ijic.10214)
Supplement: Supplementary file 6 [file ijic-26-3-10214-s6.pdf]

SUPPLEMENTARY FILE 6: CASE VIGNETTE RESULTS

Table S6.1. Collected case vignettes and key results

No	Case name	Key words	Referral decision expert 1*	Referral decision expert 2*	Considerations and dilemmas	Missing information
1	Mr Acacia	Symptoms<br>Collaps, abdominal pain, nausea and vomiting, weight loss, hypotension<br>Diagnosis<br>Vasovagal syncope, acute on chronic kidney injury, suspected urinary tract infection<br>Treatment<br>AB IV, IV fluids<br>Care challenges<br>Risk of malnutrition	AGCH	AGCH	• If outpatient clinic is offsite, short hospital readmission may be required to insert a PICC line<br>• Advanced imaging might be needed, which is difficult outside hospital	• n/a
2	Ms Carnation	Symptoms<br>Seizure, weight loss, functional decline,<br>Diagnosis<br>Hypoglycemia, mild hypercalcemia, suspected depression<br>Treatment<br>Blood glucose regulation, insulin adjustment, monitoring of vital signs<br>Care challenges<br>Mobility impaired, cognitive decline, caregiver burden, limited informal support	STCR/GR	STCR/GR - AGCH	• Unclear if direct discharge home is feasible due to limited information<br>• Admission can occur in short-stay nursing home unit (no outpatient clinic needed)	• Medical history, support network, home care, and medication reasons unclear<br>• Cognitive status unknown, especially important if depression suspected
3	Mr Sunflower	Symptoms<br>Dyspnoe, mild cognitive impairment, hypotension<br>Diagnosis<br>COPD exacerbation, delirium, heart failure, pneumonia, hypoglycemia, acute kidney injury<br>Treatment<br>Vital signs every 8 hours, daily weight and fluid balance, daily lab tests, IV medication, IV antibiotics<br>Care challenges<br>Mild cognitive impairment, functional decline	Hospital	Hospital	• Oxygen at 6L, IV Lasix, and IV antibiotics likely too complex for outpatient care<br>• Hospital admission is warranted, since the patient was digitized and cardiology was consulted regarding possible inotropes	• Complete medical history and GP contact status unclear without support system<br>• Clarification of overarching treatment goals needed
4	Mr Hibiscus	Symptoms<br>Hypotension, tachycardia, weight loss, cognitive decline<br>Diagnosis<br>Urosepsis, acute on chronic kidney insufficiency, recurrent urinary tract infections dementia<br>Treatment<br>IV antibiotics, IV fluids, monitoring vital signs, fluid balance monitoring<br>Care challenges<br>Delirium, dementia, increased risk of falls, malnutrition, functional decline and pressure ulcers	AGCH	AGCH - Hospital	• Admission to outpatient clinic is appropriate given the patient's dementia, current delirium, and previously good functioning at home	• Severity of kidney dysfunction and delirium unknown; symptoms unspecified
5	Mr Mimosa	Symptoms<br>Dyspnoea, orthopnea, low oxygen saturation, hypotension, acute deterioration, generalized edema<br>Diagnosis<br>Heart failure, pneumonia, acute on chronic kidney insufficiency<br>Treatment<br>IV medication, oxygen therapy, oral antibiotics, eventually emergency transfer to hospital<br>Care challenges<br>Risk of acute deterioration	AGCH	AGCH - Hospital	• Reason for ambulance transfer back to hospital is unclear<br>• If a maximum conservative treatment plan was in place, symptom relief might be manageable in the outpatient clinic<br>• If patient still considers interventions like NIV, outpatient	• Pre-existing cardiac function is unclear.

					clinic may be unsuitable	
6	Mr Poppy	Symptoms<br>Dyspnea, fever, and general malaise, coughing, vomiting, mild cognitive deterioration<br>Diagnosis<br>Pre-existent prostate cancer with osseous metastases, Influenza A, acute-on-chronic kidney insufficiency, normocytic anemia,<br>Treatment<br>Oxygen therapy, IV fluids, IV antibiotics<br>Care challenges<br>Delirium, risk of functional deterioration, limited informal support network, caregiver overburden	STCR/GR - AGCH	AGCH	n/a	• Unclear why IV antibiotics chosen over oral; oral may suffice for standard recovery<br>• Vomiting considered alongside hypercalcemia due to prostate cancer metastases<br>• Unclear if hypercalcemia was confirmed or tested
7	Ms Daffodil	Symptoms<br>Dyspnea, coughing, fatigue and weakness, known chronic pain, reduced mobility, mild hyponatremia<br>Diagnosis<br>Influenza A, microcytic anemia, acute decompensated heart failure after packed cells<br>Treatment<br>Oxygen therapy, nebulization, one unit packed cells<br>Care challenges<br>Limited mobility, limited social support, grief, possible mild cognitive impairment	AGCH	AGCH	• Case complexity depends on degree of decompensation, which is currently unclear	• Medication use and glucose levels missing; symptoms and treatment after decompensation need clarification
8	Mr Orchid	Symptoms<br>Cognitive impairment, recurrent falls, low body weight, incontinence, signs of delirium, poor nutritional state<br>Diagnosis<br>Urinary tract infection, hyperglycemia, suspected vitamin D deficiency, recurrent falls<br>Treatment<br>IV antibiotics, adjustment of diabetes medication<br>Care challenges<br>High risk of wandering behavior, diabetes dysregulation, and sepsis, Limited social support, delirium and dementia.	STCR/GR	AGCH	• Risk of treatment failure due to potential IV removal by patient	• More info needed on home care situation and cause of low body weight<br>• Physical exam and tests required to assess clinical severity<br>• Cause of recurrent falls unknown
9	Ms Gerbera	Symptoms<br>Moderate nutritional status with insufficient and unbalanced intake, reduced mobility developing over past months, tendency to fall<br>Diagnosis<br>No acute diagnosis on presentation<br>Treatment<br>Continued observation and further diagnostics as needed<br>Care challenges<br>Lonelyness, fall risk, risk of delirium, risk of physical deconditioning	STCR/GR - AGCH	AGCH	• Not eligible for AGCH due to no intercurrent medical diagnosis and no need for low-complexity hospital-level treatment	• Unclear how patient was mobilizing at home before ER visit
10	Ms Buttercup	Symptoms<br>Psychosis and delusions, automutilation of right side of face, urinary retention, decreased food intake over last days<br>Diagnosis<br>Delirium with psychotic features, mild hyponatremia, urinary tract infection, urinary retention<br>Treatment<br>Indwelling catheter, IV antibiotics, fluid restriction and close monitoring due to psychosis and delusions<br>Care challenges<br>Risk of wandering or leaving care area, risk of further automutilation, lack of social support	AGCH	STCR/GR	• Possible underlying psychiatric issues complicate AGCH care due to limited psychiatric access	• Clarification needed on medication, previous delirium episodes, and patient's home activity level

11	Ms Lotus	Symptoms<br>General malaise, fever, cough, weakness and fatigue causing decreased mobility, reduced food intake past 4 days<br>Diagnosis<br>COVID-19, chronic kidney insufficiency, Normocytic anemia<br>Treatment<br>Monitoring symptoms, oxygen therapy when needed, IV fluids<br>Care challenges<br>Risk of functional decline, patient tends to understate symptoms, risk of caregiver burden	AGCH	STCR/GR - AGCH	• Without IV, referral would typically be via ER chain to rehab or nursing home, not AGCH	• Need for saline IV unclear<br>• More info needed on patient's support network
12	Mr Anemone	Symptoms<br>Dyspnea, subfebrile temperature, cough, frequent falls with back pain and minor head trauma, decreased appetite and poor nutritional intake (recent days)<br>Diagnosis<br>COVID-19 infection with bacterial superinfection, delirium, atrial fibrillation with rapid ventricular rate chronic kidney dysfunction, urinary retention, asymptomatic hyperkalemia<br>Treatment<br>Oral antibiotics (later switched to IV due to deterioration), start indwelling catheter, IV fluids, EWS monitoring<br>Care challenges<br>Delirium, fall risk, functional decline, patient is care avoiding, social isolation, coping difficulties	AGCH	STCR/GR - AGCH	• AGCH may struggle to care for severely confused patients during evenings and nights<br>• If condition worsens the dilemma is wether to treat in the nursing home or hospital	• Unclear if active treatment plan exists, including hospital interventions, symptom management, and ICU eligibility
13	Ms Lily	Symptoms<br>Downward spiral due to vertebral compression fractures, back pain, constipation: no bowel movement for 7 days<br>Diagnosis<br>Osteoporose with two new compression fractures, left rib-tip syndrome, decubitus ulcer grade 2, constipation.<br>Treatment<br>Laxatives administration, DEXA scan, pain monitoring, treatment of pressure ulcer<br>Care challenges<br>Fall risk, risk of pressure ulcers, deterioration, risk of functional decline	STCR/GR	STCR/GR	• Requires regular geriatric rehabilitation: physical therapy, occupational therapy, dietetics	• Patient's treatment wishes are unclear
14	Mr Amaryllis	Symptoms<br>Acute general malaise, diarrhea, vomiting, decreased mobility<br>Diagnosis<br>Gastroenteritis. During admission prostatitis (diagnosed by urology following difficult and traumatic catheter insertion after urinary retention)<br>Treatment<br>IV fluids, IV antibiotics, stool culture, EWS three times a day.<br>Care challenges<br>Nutritional deterioration, functional decline, risk of delirium, high infection risk (urinary catheter complications)	AGCH	AGCH	• Somatic treatment plan present, but no overall recovery plan<br>• Question if current interventions align with patient's goals and wishes	• Overarching treatment goals and personal wishes are missing
15	Mr Daisy	Symptoms<br>Generalized weakness; unable to get up at home due to loss of leg strength, disorientation<br>Diagnosis<br>Suspected urinary tract infection or upper respiratory tract infection, delirium, fall during admission (no intracranial bleeding on CT)<br>Treatment<br>IV antibiotics, EWS 3 times a day, observation of cognitive status.<br>Care challenges<br>Fall risk, functional decline, suspected dementia, the patient is care-avoidant,	STCR/GR	STCR/GR	• If returning home is not possible, care challenges will arise quickly<br>• Outpatient follow-up currently seems feasible<br>• Alternative: geriatric specialist consultation in primary care, including home visits	• Clarify if care is short-term recovery or long-term<br>• More info needed on cognitive functioning<br>• Clear treatment goals and wishes needed, aligned with patient's identity

		non-adherent to medication, and has poor illness insight due to cognitive decline.				
16	Mr Gladiolus	Symptoms<br>Poor appetite and recent weight loss, mobility severely reduced, unable to get out of bed at home, walking difficult and refused rollator, cognitive impairment<br>Diagnosis<br>COVID-19 infection, kidney insufficiency, delirium, hypertension, Early dementia suspected (not yet diagnosed)<br>Treatment<br>IV antibiotics, IV fluids, nutritional support, EWS three times a day<br>Care challenges<br>Fall risk, delirium, suspected cognitive impairment affecting medication adherence and self-care risk of malnutrition	AGCH	AGCH	• Short-stay nursing home could be an option if IV antibiotics aren't needed	• Reason for IV antibiotics and fluid bolus is unclear
17	Mr Poinsettia	Symptoms<br>Fever and general malaise, vomiting, decreased appetite, weight loss, confusion, leg weakness, dehydration<br>Diagnosis<br>Fever without clear focus, clinical deterioration with increased infection markers, hypotension, suspected worsening infection during admission requiring hospital transfer<br>Treatment<br>IV fluids, IV potassium supplementation, IV antibiotics, nutritional support, imaging X-thorax and CT-scan<br>Care challenges<br>Fall risk, risk of functional decline, risk of delirium, risk of non-adherence to treatment	AGCH	AGCH	• Diagnosis unclear at AGCH admission, making clinical course unpredictable<br>• Performing CT scan in AGCH seems inappropriate<br>• Extensive monitoring needed, depending on AGCH capacity	• Diagnosis unclear at AGCH admission, making clinical course unpredictable<br>• Longstanding physical and functional impairment without clear explanation; duration unspecified
18	Mr Dandelion	Symptoms<br>General weakness, impaired mobility, hallucinations and fluctuating consciousness, constipation<br>Diagnosis<br>Urinary tract infection, de novo atrial fibrillation (AF), constipation, general weakness related to infection and Parkinson's disease progression, delirium<br>Treatment<br>IV antibiotics, laxative treatment, delirium management, anticoagulation therapy<br>Care challenges<br>Fall risk, delirium, lack of insight complicating care adherence, rapid clinical deterioration	STCR/GR - AGCH	AGCH	• Is 8-day recovery after UTI realistic for mobilization and delirium resolution?<br>• Treat UTI with IV antibiotics or manage via ER plus short-stay nursing home?	• Physical exam missing (e.g., edema, ventricular rate in AF)<br>• Additional tests needed (blood work) to assess severity<br>• Heart failure noted, but no clear management plan<br>• Missing info on current medication and kidney function
19	Ms Violet	Symptoms<br>Found on the floor, decreased responsiveness, confused behavior, urinary retention, dehydration, pain (related to vertebral compression fracture)<br>Diagnosis<br>Urinary retention with urinary tract infection, dehydration, new vertebral compression fracture at level L2, rhabdomyolysis, kidney insufficiency, tachycardia and hypotension<br>Treatment<br>IV fluids, IV antibiotics, urinary catheter placement, pain management, close monitoring EWS<br>Care challenges<br>Possible deterioration, limited social support, risk of further functional decline	AGCH - Hospital	AGCH	• Questionable if 8-day recovery with adequate mobility and self-care for home discharge is realistic<br>• Patient clinically unstable at AGCH admission (hypotension, tachycardia)<br>• Monitoring these parameters in AGCH may be insufficient	• No physical exam or additional diagnostics reported except CT scan
20	Mr Tulip	Symptoms<br>Dyspnea, general weakness, mild cognitive impairment<br>Diagnosis<br>Heart failure, hyponatremia, hypokalemia, suspected infection (elevated CRP)<br>Treatment	Hospital	AGCH	• Heart failure without clear cause; echocardiogram during admission may be needed<br>• IV diuresis with hypokalemia	• Heart failure noted, but cardiac background unclear<br>• Severity of hypokalemia unknown<br>• Patient's clinical status unclear;

	IV diuretics, urinary catheter placement, potassium supplementation, laboratory monitoring, daily weighing, close monitoring electrolyte imbalance<br>Care challenges<br>Risk of dehydration, cognitive impairment, risk of functional decline, hearing impairment, limited social support, fall risk		risk; telemetry might be required	oxygen requirement unknown<br>• DNR order in place; unclear if NIV needed or situation is mild<br>• Uncertain if NIV/ICU options still considered<br>• No medication list provided<br>• Diagnosis of MCI/dementia unclear
