# Supplementary material for: Two Dutch Examples of Acute Geriatric Community Hospitals for Older Adults: A Comparative Process Evaluation
Source: Int J Integr Care. 2026 Sep 8;26(3):18. doi: 10.5334/ijic.10214 (PMC13564377; doi:10.5334/ijic.10214)
Supplement: Supplementary file 7 [file ijic-26-3-10214-s7.pdf]

SUPPLEMENTARY FILE 7: FOCUS GROUP INTERVIEW GUIDE

Care Phase	Questions
Introduction	-
Referral	How is triage conducted at the ED?<br>How was it assessed whether admission to the AGCH was appropriate?<br>How is information about available beds in the AGCH shared with the ED?<br>How often do you have to refuse admissions due to full capacity?<br>Are patients often rejected for admission to the AGCH? If yes, why?<br>How are patients and their relatives involved in the decision to admit to the AGCH?<br>What processes are in place at the ED to ensure smooth triage to the community clinic?<br>How do you think the referral process generally goes?<br>What are common points of attention or improvements opportunities in the refferal phase?
Admission preparation and transfer	What does the admission preparation process involve?<br>How is the patient and/or his relatives informed about the AGCH?<br>Who handled the medical handover from the ED to the AGCH, and how?<br>How does the AGCH team prepare for a patients arrival (non-medical)?<br>Who coordinated the timing of the transfer to the AGCH?<br>Who created the patient file? What information was added to the patient file and how?<br>Who was responsible for preparing the patient room and materials?<br>Are there other preparations needed before a patient arrives?<br>Are the patient and/or his relatives expected to take any action before the transfer to the AGCH?<br>How do you think preparation for admission and transfer generally goes?<br>What are common points of attention or improvement opportunities in the preparation and/or transfer phase?
Admission	What does the arrival and initial reception of a patient in the AGCH look like?<br>What other matters were arranged or scheduled for the patient immediately after arrival?<br>Who was responsible for this?<br>How do you think the arrival of patients generally goes?<br>What are common points of attention or improvement opportunities in the arrival phase?
Intake	Who is present during the admission interview?<br>What does the intake process involve?<br>Is a Comprehensive Geriatric Assessment (CGA) conducted? If yes, by whom and how?<br>Is a physical examination and/or clinimetrics performed? If yes, by whom and how?<br>Is a medication review conducted? If yes, by whom and how?<br>Are treatment goals and limitations discussed? If yes, by whom, with whom, and how?<br>Is the patient's care network mapped or assessed? If yes, by whom, with whom, and how?<br>Is the patient's social network mapped or assessed? If yes, by whom, with whom, and how?<br>Is advance care planning being carried out? If yes, by whom, with whom, and how?<br>Is additional information requested from the patient's care network? (e.g., GP, district nurse, home visit for housing assessment?)<br>When and by whom were the intake and clinimetric results entered into the patient record?<br>When and by whom were the intake and clinimetric results reviewed or evaluated?<br>How do you think the intake of patients generally goes?<br>What are common points of attention or improvement opportunities in the intake phase?
Treatment	Which disciplines are involved in treatment and how intensively?<br>What is the general structure of the multidisciplinary meetings? (e.g., format, number of patients discussed, attendees)<br>When and how is feedback given to the patient and/or his relatives about the multidisciplinary meetings?<br>Who is responsible for creating the care pathway, entering the estimated discharge date, setting the number of treatment hours per week, and opening the funding?<br>What measures are taken to address the risk of functional decline?<br>What fall prevention strategies are implemented?<br>What delirium prevention measures are in place?<br>What is done to address the risk of malnutrition?<br>Is there a possibility for rooming-in?<br>How do you think the treatment of patients generally goes?<br>What are common points of attention or improvement opportunities in the intake phase?
Discharge preparation	When does the discharge preparation phase begin?<br>What does the discharge preparation process generally look like?<br>Is a second multidisciplinary meeting (MDM) always scheduled for the patient? If not, how is discharge preparation coordinated within the team?<br>When, and by whom, is the patient and/or their relatives informed about the final discharge date, destination, and any further care arrangements?<br>What matters need to be arranged before a patient can be discharged?<br>Who was contacted for a 'warm' handover and by whom?<br>How was this contact made?<br>What information was exchanged?<br>How do you think the discharge preparation of patients generally goes?<br>What are common points of attention or improvement opportunities in the discharge preparation phase?
Discharge	Is a discharge conversation held with each patient? If so when, by whom, and who was present?<br>What information do patients and/or their relatives receive immediately before and/or after the discharge conversation? How was this information communicated?<br>After a patient is discharged, who informs whom that a bed is available and needs to be prepared for the next admission?<br>How do you think the discharge of patients generally goes?<br>What are common points of attention or improvement opportunities in the discharge phase?
Additions	What did you think of this focus group? Do you have any feedback for us?<br>Did this focus group spark any improvement ideas?<br>What improvement goal is connected to this ideas, and how could we measure it?<br>Who could do what, and by when, to achieve this improvement goal?
