# Supplementary material for: Two Dutch Examples of Acute Geriatric Community Hospitals for Older Adults: A Comparative Process Evaluation
Source: Int J Integr Care. 2026 Sep 8;26(3):18. doi: 10.5334/ijic.10214 (PMC13564377; doi:10.5334/ijic.10214)
Supplement: Supplementary file 8 [file ijic-26-3-10214-s8.pdf]

SUPPLEMENTARY FILE 8: CODE TREE

- Core components
    - Personalised, specialistic, acute geriatric care
        - CGA
        - Intake
        - Medication review
        - ACP
        - Continuous monitoring
        - Adequate response
        - Diagnostics
        - Treatment
    - Integrated care focused on returning home
        - Interdisciplinary team
        - Multidisciplinary team meeting
        - Coordination within the healthcare network
        - Discharge (planning)
        - Involvement family and caregivers
    - Prevention in areas of vulnerability
        - Screening for geriatric syndromes (functional decline, falls, delirium, malnutrition)
        - Implementing interventions (functional decline, falls, delirium, malnutrition)
    - Appropriate environment close to home
        - Stimulus-reduced environment
        - Recovery-oriented climate
        - Visiting hours and rooming-in
        - Space for social contact

- Referral appropriateness
    - The patient has an acute medical care need.
    - The patient has one or more geriatric syndromes.
    - The medical specialist must be involved in the treatment.
    - The patient is hemodynamically stable.
    - The diagnoses and treatment plan are clear before AGCH admission.
    - The required treatment (complexity) is possible at the AGCH.
    - The diagnostic tests needed to monitor the effectiveness of treatment are available at the AGCH.
    - The required continuous nursing assistance and monitoring intensity is feasible at the AGCH.
    - Risks (of treatment) lead to consequences which are treatable at, or in close proximity to, the AGCH (or the patient has a treatment limitation policy such as no intensive care).
    - In case of treatment of patients with wandering symptoms, behavioural problems and infectious diseases (e.g. influenza, COVID), safe admission to the AGCH is possible.
    - Return to the previous living situation (or admission to residential LTC) is expected within 14 days.
    - Early rehabilitation and activation to prevent functional decline will benefit the patient
    - A low stimuli environment will benefit the patient (e.g. delirium preventive)

- Improvement opportunities
    - Appropriate referral
        - Challenges related to appropriate referral
        - Solutions to improve appropriate referral
    - Managing expectations
        - Challenges related to managing expectations
        - Solutions to improve managing expectations
    - Discharge planning
        - Challenges related to discharge planning
        - Solutions to improve discharge planning
    - IT system integration
        - Challenges related to IT system integration
        - Solutions to improve IT system integration
    - Structural funding
        - Challenges related to structural funding
    - Care during evening, nights and weekends
        - Challenges relate to care during evening, nights and weekends
        - Solutions to improve care during evening, nights and weekends
